# Highlighting radical sites through polarized neutron scattering from AFP-modulated polarized protons

**DOI:** 10.1107/S2052252525005871

**Published:** 2025-08-21

**Authors:** Patrick Hautle, Oliver Zimmer, Hélène M. Jouve, Heinrich B. Stuhrmann

**Affiliations:** a Paul Scherrer Institute 5232Villigen Switzerland; b Institut Laue–Langevin 38000Grenoble France; c Institut de Biologie Structurale 38000Grenoble France; d Helmholtz-Zentrum Hereon, 21494Geesthacht, Germany; Chinese Academy of Sciences, China

**Keywords:** polarized neutron scattering, dynamic polarized protons, adiabatic fast passage, tyrosyl radical, bovine liver catalase, structural biology, solution scattering, time-resolved studies, charge, spin and momentum densities, dynamical simulations

## Abstract

Time-resolved neutron scattering has been used to study dynamically polarized protons in tyrosyl-doped bovine liver catalase. While the evolution of proton polarization and its inversion by the method of adiabatic fast passage (AFP) in a standard dynamic nuclear polarization (DNP) system with organic Cr(V) complexes can be well modelled and understood, the experiments with tyrosyl-doped catalase lead us into the world of extremely dilute paramagnets with only about 10^17^ unpaired electrons per cm^3^. In this regime, the strength of DNP is comparable to the drift of proton polarization towards its thermal equilibrium of *P*_e_ = 0.35% at *T* = 1 K and *B* = 3.5 T. Negative DNP, which counteracts this drift, is confined to protons very close to the radical site, typically within 5 Å. In contrast, AFP reverses the polarization of protons at a larger distance from the radical. The contrast of a domain of polarized close protons giving rise to neutron scattering is considerably enhanced by AFP. Moreover, the spread of proton polarization is sensitive to magnetic inhomogeneities, like the iron of the heme of the catalase molecule. In short, polarized neutron scattering from AFP-modulated polarized samples provides an excellent tool for mapping sources and sinks of proton polarization in radical proteins.

## Abstract 2

Selective nuclear spin reversal by the method of adiabatic fast passage is a way to order a system of dynamically polarized nuclei. Using polarized neutron scattering, it increases the visibility of sources and sinks of proton polarization in radical proteins. Notably, Tyr369 has been confirmed as a potential radical site in bovine liver catalase.

## 1. Introduction

It was at a symposium on radical proteins at the chateau of Sassenage near Grenoble in September 1997 when one of us, HBS, listening to the lecture given by HMJ, noticed a striking similarity of the EPR line of the catalase-bound tyrosyl radical with that of an organic chromium-V complex routinely used for strongly polarized proton spin targets in high-energy physics, for instance Niinikoski (2020 ▸). Could the tyrosyl radical inside the catalase molecule do the same job?

Using his best French, HBS dared to draw the attention of HMJ to a possible localization of the tyrosyl radical site, referring to a well known paper from Hayter *et al.* (1974 ▸). Quite to his surprise and also to his great relief, *Madame* understood immediately. The nucleus of a common project was defined in less than 10 minutes, just before lunchtime. Would it find the badly needed support in the scientific community?

Once more, it is a good idea that helps. Protein bound radicals potentially supporting dynamic nuclear polarization (DNP) appealed to our Swiss friends working with polarized targets at the Paul Scherrer Institute, Villigen. Their competence in the use of NMR techniques, like adiabatic fast passage (AFP) for the inversion of nuclear polarization, was to become a crucial element of our project. The rumour about this initiative did not escape Hans Glättli (CEA Saclay), who put the focus on the evolution of nuclear polarization at the onset of microwave irradiation of free-radical molecules. Studying the change of nuclear polarization with time in the light of polarized neutron scattering was a real turning point. Last but not least, physicists from the neutron scattering community also joined, like Oliver Zimmer from the Technical University Munich, now at the Institut Laue–Langevin, Grenoble. The complete list of our collaboration can be found in van den Brandt *et al.* (2002 ▸, 2004 ▸, 2006 ▸, 2007 ▸).

Polarized neutron scattering from dynamic polarized protons of a radicalized protein raises a number of questions concerning its feasibility. Assuming significant support of DNP by tyrosyl radicals, an extremely small number of these radicals among several tens of thousands of atoms of catalase might still pose serious problems with respect to the outcome. We agreed on time-resolved neutron scattering of small radical molecules like an organic complex of Cr(V) of 10 Å diameter (*M* = 328) and a number of medium-sized radical molecules up to 40 Å length (*M* ≤ 1000) to start with. The doubts concerning the so-called magnetic nuclear spin diffusion barrier could be alleviated. The sequence of alternating directions of DNP provided the key for the analysis of the AFP-modulated proton polarization, which had been done in March 2000.

As promising as this project looked, our enthusiasm was not shared, even by the pioneers of polarized neutron diffraction from dynamic polarized protons in a single crystal of lanthanum magnesium nitrate. In 1983, John Hayter, mentioned above, wrote to HBS in a personal letter that, rather than using NMR/ESR methods to achieve high proton polarization, he would rely on brute-force proton polarization, instead. Higher magnetic fields and much lower temperatures could provide stable and uniform proton spin targets more suitable for crystallographic studies in spite of their modest polarization.

Our strategy differed fundamentally from the approach of John Hayter in two ways:

(1) Accept the changeability of proton polarization in time-resolved experiments.

(2) Accept the random orientation of particles under consideration in small-angle scattering.

At that time, DNP with the help of paramagnetic impurities developed rapidly. Usually, the latter were dissolved in antifreeze solvents. Many of them, like glycerol, were perfectly compatible with biomolecules. Thus, we had a large choice of samples suitable for time-resolved polarized neutron small-angle scattering from dynamic polarized protons. This was the renaissance of the method of nuclear spin contrast variation which started in Europe and particularly in Japan, where it is nowadays most actively developed in materials science.

In a final step, and almost accidentally, as mentioned above, we came across a paramagnetic centre as a native constituent of an enzyme present in many living systems and which is intimately related to its function, the splitting of hydrogen peroxide into water and molecular oxygen by bovine liver catalase (BLC). The structure of BLC had been determined by Fita & Rossmann (1985 ▸). The way to unravel some aspects of its function focused on the migration of proton polarization inside the catalase molecule triggered by DNP and AFP. Our approach is intrinsically a dynamical one which works on a platform provided by X-ray crystallography. To make a long story short, let us go into *medias res*, the DNP.

## 2. Creation of proton polarization by DNP and its reversion by AFP

Dynamic nuclear polarization is a means to achieve high proton polarization in hydrogenous material, typically by prolonged microwave irradiation. The presence of a small quantity of paramagnetic impurities, unpaired electrons for instance, is mandatory. The sample is kept in a strong magnetic field strength, *B* > 2 T, at temperatures below or close to 1 K. The polarization of the electrons is then almost complete. In fact, it is the high polarization of an electron spin system which is transferred to the nuclear spin system by microwave irradiation exploiting the dipolar interaction of the systems (Abragam & Goldman, 1978 ▸, 1982 ▸; Glättli & Goldman, 1987 ▸). At the onset of microwave irradiation, proton polarization by DNP starts in the near vicinity of an unpaired electron. In a next step the polarization diffuses into the bulk.

This experiment has been carried out using a dynamic polarization facility operated at a temperature of 1 K in a magnetic field of 3.5 T. It has been developed at the Paul Scherrer Institute (PSI) at Villigen, Switzerland, and modified for neutron scattering experiments (van den Brandt *et al.*, 2002 ▸). A 3.5 T split coil wound on an aluminium former is attached to the bottom of a liquid ^4^He vessel. A 49 mm diameter stainless steel tube with an aluminium end cap runs axially through the helium bath and then under vacuum down to centre of the magnet. It accommodates a continuous flow ^4^He refrigerator insert with a top loading sample holder device. The setup allows a quick sample change.

**Figure 1:**
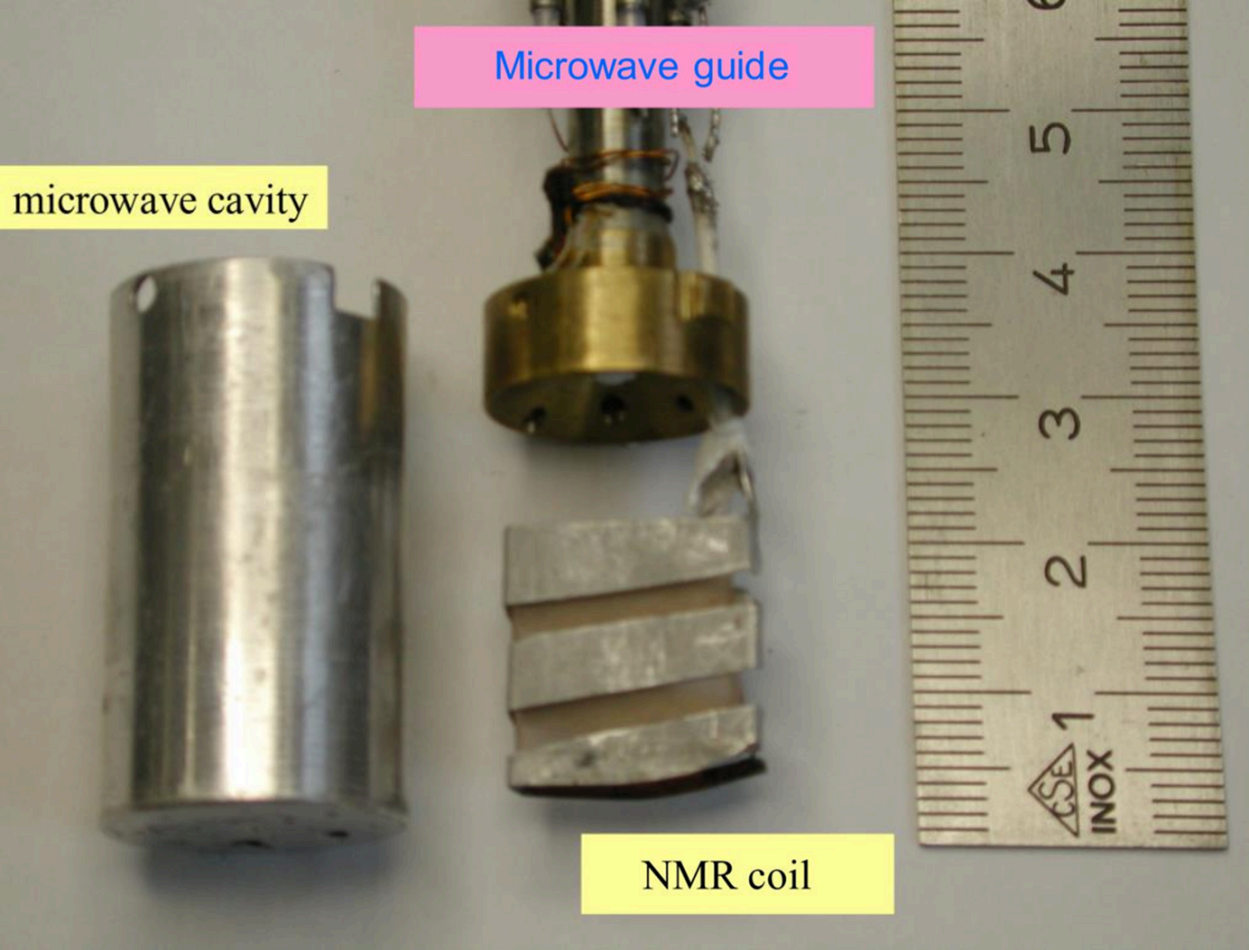
Solid samples are inserted into the NMR coil, with the microwave cavity fitting around it. Microwaves enter the cavity through the guide at the top.

The samples under investigation were glassy slabs, prepared by injecting the solution into a copper mould cooled with liquid nitro­gen. These samples were inserted into the NMR coil placed inside the microwave cavity (Fig. 1 ▸). For DNP, an IMPATT diode provided the microwave frequency of around 97 GHz, while a continuous-wave NMR system monitored the bulk proton polarization. Further details on the DNP apparatus can be found in van den Brandt *et al.* (2004 ▸).

The direction of proton polarization depends on the choice of the microwave frequency close to the EPR line of the unpaired electron. DNP will proceed in the positive direction at frequencies below the EPR line and it will drive the proton polarization to negative values at frequencies above the EPR line. In a field of 3.5 T the EPR line for our samples is close to 97.35 GHz.

Now we turn to the NMR coil. In a magnetic field of 3.5 T it is operated at a radio frequency RF = 148 MHz. An RF sweep across the NMR line is used for both the measurement of the proton polarization and the reversal of the proton polarization. It is the power of the RF and the sweep speed that make the difference. A fast sweep of a few milliseconds with very low power, which hardly affects the proton polarization, is used for the measurement of the latter with the so-called *Q*-meter method (Court *et al.*, 1993 ▸). A much higher RF field amplitude and a slower sweep of typically 0.3 s are required to achieve the condition of adiabaticity, resulting in an efficient reversal of the proton polarization (Bloch, 1946 ▸; Hautle *et al.*, 1992 ▸, 1995 ▸).

**Figure 2:**
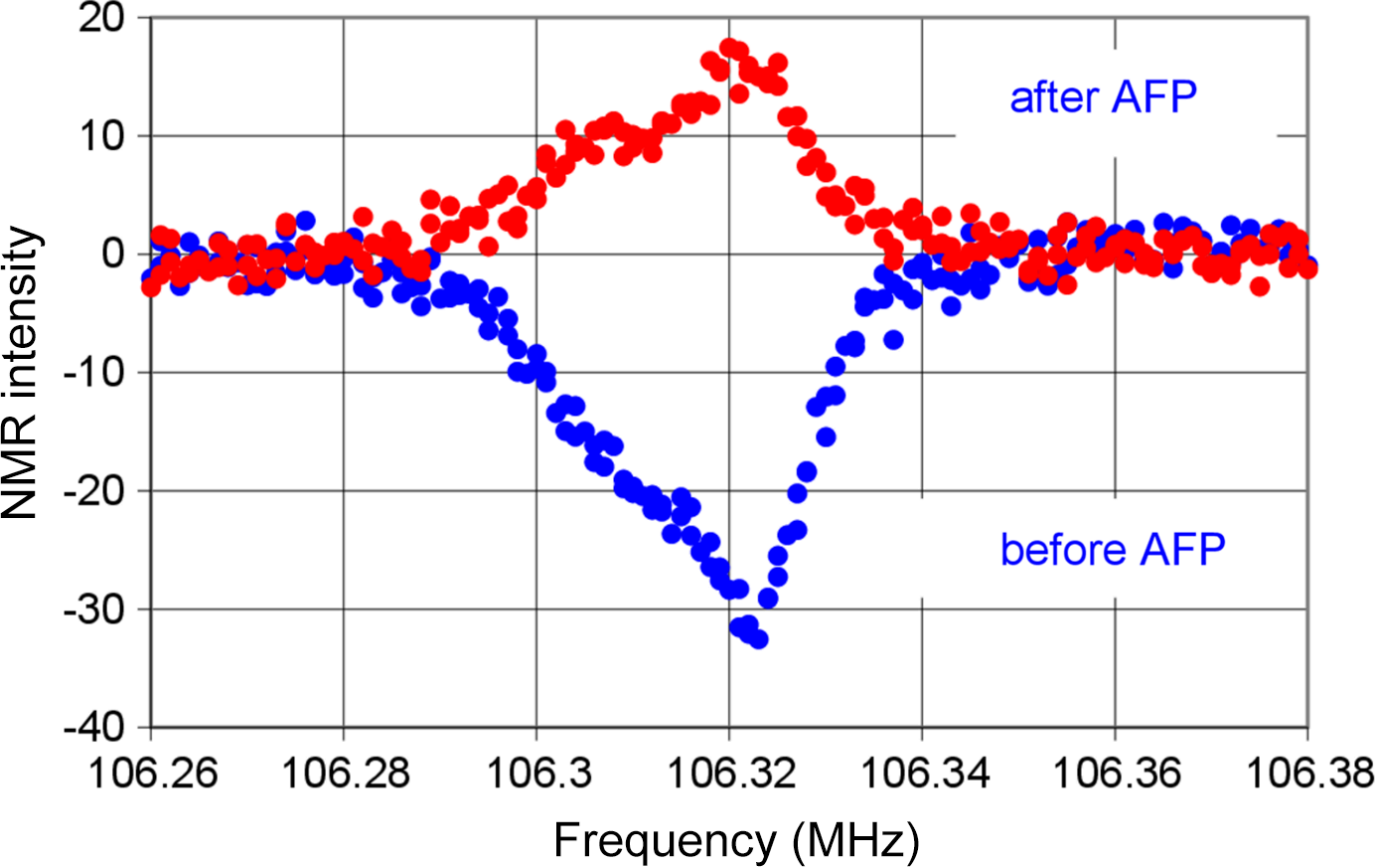
Proton NMR profile of tyrosyl-doped catalase before and after AFP. The efficiency of AFP is ɛ = 0.50. The sample was kept under a magnetic field of *B* = 2.5 T at a temperature of *T* = 0.5 K. Note that with both NMR and AFP methods, the RF field is swept across the NMR line, primarily affecting protons located outside the local field produced by paramagnetic impurities.

We define the efficiency ɛ of AFP as
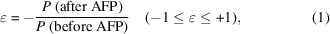
where ɛ may have values between −1 and +1. Complete inversion of the polarization by AFP is characterized by ɛ = 1. ɛ = 0 means complete depolarization. With ɛ = −1, AFP does not change the polarization. An example of an AFP polarization reversal with ɛ = 0.5 is given in Fig. 2 ▸.

The calibration of NMR intensity in units of proton polarization is performed under thermal equilibrium conditions, at a temperature of 1 K in a magnetic field strength of *B* = 3.5 T, where the proton polarization is 0.35%.

In this paper, we introduce how the efficiency of the AFP differs between various reservoirs due to the same underlying mechanism that causes the different evolution of proton polarization. The local magnetic fields generated by the paramagnetic centres cause significant shifts in the Larmor frequencies of nearby protons.

During AFP, an RF field is swept across the NMR line, as shown in Fig. 2 ▸. However, the proton spins that contribute most significantly to the observed NMR signal originate primarily from the bulk – those not strongly perturbed by local magnetic fields. In contrast, the protons located close to the paramagnetic centres experience broad spectral shifts, extending to several megahertz or more depending on their distance from the centre. Consequently, only a small fraction of these spins is affected by the RF sweep, resulting in a lower overall efficiency for these.

Relaxation during AFP (relaxation in the rotating frame) can influence the efficiency of the process. However, for a given spin system, the RF field strength and the sweep speed are chosen to minimize these effects. In our case we do not consider relaxation to be a dominant factor responsible for the different AFP efficiencies of the reservoirs.

## 3. Nuclear spin diffusion barriers

Two types of nuclear spin diffusion barriers create gradients of proton polarization within the sample:

(1) Barriers caused by abrupt change in isotopic composition.

(2) Barriers caused by magnetic inhomogeneities and paramagnetic impurities.

Previous studies on nuclear spin diffusion barriers involving radical molecules will be revisited.

### 3.1. Isotope dependent polarization gradient

In the first case, the surface of a hydrogenous molecule dissolved in a deuterated solvent separates the region of the solute rich in ^1^H from that of the solvent rich in ^2^H. Time-resolved polarized neutron scattering from dynamic polarized protons of an organic complex of Cr(V), EHBA-Cr(V), in a deuterated solvent proves the presence of a barrier due to an isotopic gradient (van den Brandt *et al.*, 2002 ▸, 2004 ▸, 2006 ▸). At the same time, preliminary results from catalase and a biradical were reported (Stuhrmann, 2004 ▸).

**Figure 3:**
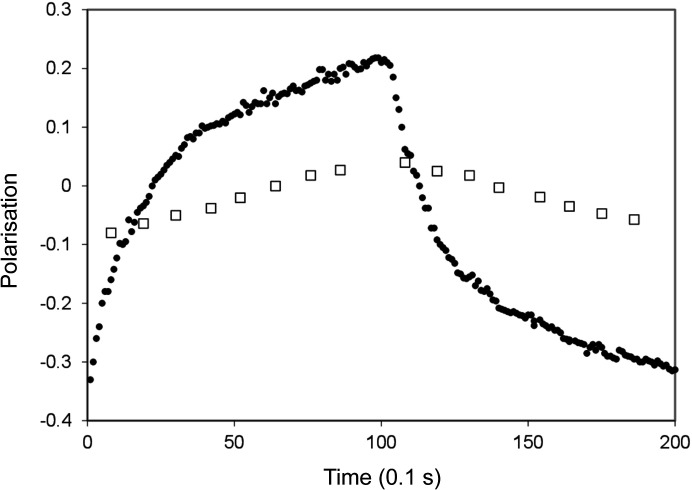
Time-resolved polarized neutron scattering (dots) and NMR (boxes) from dynamic polarized protons of EHBA-Cr(V) in a deuterated solvent. The bis­(2-hy­droxy-2-ethyl­butyrato) oxochromate anion is surrounded by 20 hydrogens of its 4 ethyl groups (Krumpolc & Rocek, 1979 ▸). Their polarization gives rise to an impressive change of the neutron scattering intensity after each change of the direction of DNP. The direction of polarization was changed every 10 s. The delayed polarization of the remote protons of the deuterated solvent seen by NMR proceeds more slowly (after van den Brandt *et al.*, 2002 ▸). The experiment was done at D22 at the ILL.

The analysis of the time-resolved measurement of both NMR and neutron scattering reveals a fast increase of the polarization of the protons of EHBA-Cr(V) followed by a slower development of the polarization of the sparce protons of the deuterated solvent, as shown in Fig. 3 ▸. While the former can be seen by the neutrons, it is the latter which gives rise to an NMR signal. The nuclear spin diffusion is controlled by the isotopic gradient at the molecular surface (Leymarie, 2002 ▸; van den Brandt *et al.*, 2006 ▸). It separates a domain R1 of protons close to the radical from the remote protons of R2 outside of the EHBA-Cr(V) molecule.

The experiments of time-resolved polarized neutron small-angle scattering were carried out at the instrument D22 of the Institute Laue–Langevin (ILL), Grenoble, France, and at the equivalent at the PSI, Switzerland. We used polarized incident neutrons of wavelengths λ = 4.6 Å with a wavelength spread of Δλ/λ = 0.1 (FWHM).

### 3.2. Intramolecular polarization gradient

As mentioned in the previous section, proton polarization by DNP develops near-paramagnetic impurities within a region that is relatively unfavourable for interactions. The Larmor frequency varies with the local magnetic field strength and this interaction impedes interactions between nuclear spins by increasing the energy difference required by ‘flip-flop’ interactions. This communication problem is the reason for the local and temporal accumulation of nuclear polarization. Finally, the nuclear spin polarization will migrate into a magnetically calmer region where the flip-flop interactions between nuclear spins occur more easily due to lower energy exchange requirements. The polarization has crossed a magnetic nuclear spin diffusion barrier.

Intramolecular gradients of proton polarization are expected in the close vicinity of a radical site. The protons close to the radical site typically within a sphere of 1 nm diameter define a region R1. The residual protons of the solute molecule belong to R2. The protons of the solvent constitute R3. Neutrons respond to polarized protons in both R1 and R2, and to a small extent to the sparce protons of the deuterated solvent excluded by the volume of the solute molecules. We come back to this point in equation (5) further below. NMR sees mainly the protons of the solvent, but also those of R2. The very close protons in R1 give rise to a broad NMR signal not easily separated from the background intensity.

### 3.3. Time-resolved experiments

Time-resolved experiments of both NMR and polarized neutron scattering provide the most effective approach for distinguishing a proton polarization across the reservoirs R1, R2 and R3.

The regions R1, R2 and R3 are coupled in series forming an onion-like structure. The driving force of DNP creates a proton polarization far from equilibrium primarily in R1 and subsequently in the regions R2 and R3.

Three rate equations govern the flow of nuclear polarization between the four reservoirs R0 and R3 coupled in series.
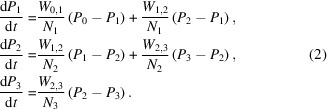
There are *N_i_* protons with the polarization *P_i_* in the reservoir *Ri*. 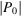 close to 1 is the polarization of the electron spin system in contact with the close protons. The rate constants 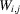 are defined as probabilities of a mutual spin flip per time unit. The solution of the three coupled linear differential equations in terms of *P*_1_(*t*), *P*_2_(*t*) and *P*_3_(*t*) is obtained by numerical methods simulating the flow of proton polarization between the four reservoirs.

After equation (7.52) in Abragam & Goldman (1982 ▸), the master equation of time-resolved neutron scattering is written as
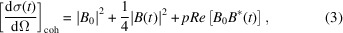
with
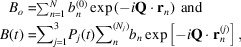
where *Q* is the momentum transfer and *r_n_* are the coordinates of the *n*th nucleus. The coordinates of the hydrogen atoms of the *j*th reservoir are denoted by 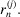 The polarization of the protons, *P*(*t*), enters into *B*(*t*) and its absolute square. The polarization *p* of the incident neutron beam only appears in the cross-term. The scattering length *b_n_* = 1.456 × 10^−12^ cm for polarized protons greatly exceeds 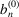 of other nuclei.

For randomly oriented particles the calculation of the intensity of coherent scattering assumes a more elegant form with the expansion of 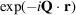 as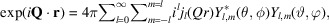 with
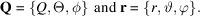
The intensity of small-angle scattering *I*(*Q*, *t*) in terms of a multipole expansion is
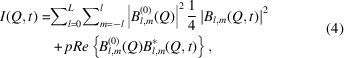
with
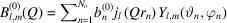
and
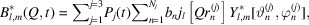
where 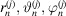 are the polar coordinates of the *n*th hydrogen atom inside the *j*th reservoir.

As the calculation of the intensity of small-angle scattering starts from amplitudes, their variation with the time-dependent proton polarization is easily implemented. Using their presentation in terms of multipoles provides elegant access to the scattering intensity from a system of randomly oriented particles.

Neglecting the square of *B*(*t*) in equation (3), the polarization-dependent time-resolved polarized neutron scattering intensity from three reservoirs can then be written as
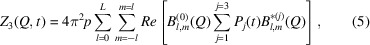
where 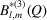 is the amplitude of the solvent protons contained inside the shape of a solute molecule. As such, its sign is negative. *P*_3_(*t*) is the time-dependent polarization of the solvent.

Using a Schärpf supermirror the polarization of the neutron beam is close to *p* = 1 (Schärpf, 1982 ▸, 1989 ▸). The index *L* terminating the expansion in terms of multipoles in equation (5) is adapted to the symmetry of the molecular structure. For the tetramer catalase molecule *L* = 4 may suffice. For spherical structures *L* = 0 will do.

The scattering function *I*(*Q*, *t*) calculated from our system of three reservoirs includes the polarization-independent part *Z*(*Q*) corresponding to the absolute square of *B_o_* [see equation (3)], the polarization-dependent intensity of both the coherent scattering *Z*_3_(*Q*, *t*) and the intensity of incoherent scattering, all of them corrected for transmission, *tr* (Zimmer *et al.*, 2016 ▸).
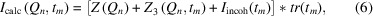
where *Q_n_* and *t_n_* are the *n*th and *m*th value of the *Q* and *t* scale, respectively. The root mean square (RMS) deviation
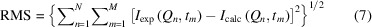
is minimized by an appropriate choice of the transition probabilities 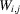 in equation (2), taking into account the (polar) coordinates of the atoms as elements of the reservoirs R1, R2 and R3.

### 3.4. The presence of intramolecular polarization gradients

**Figure 4:**
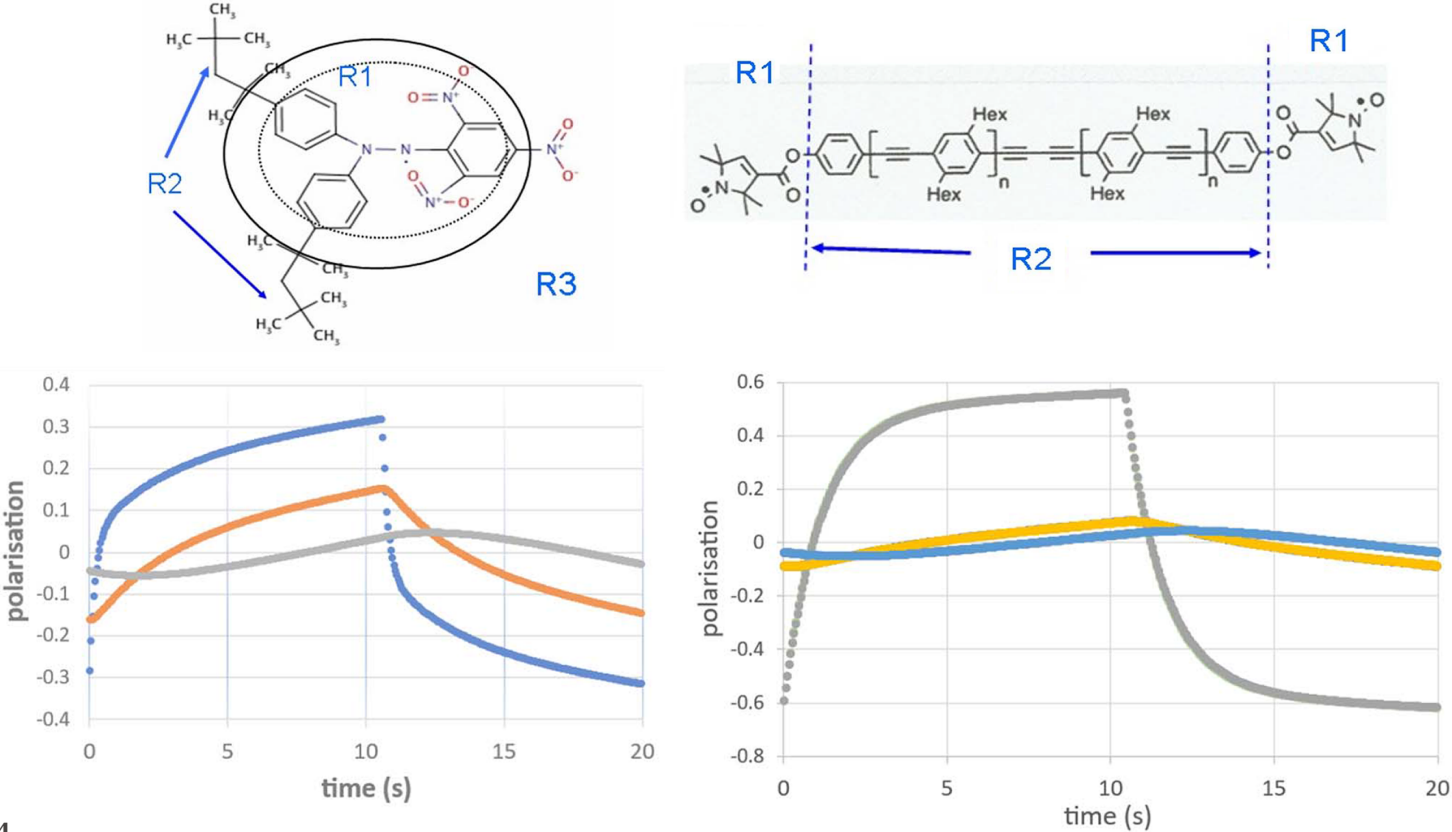
2,2-Di(4-*tert*-octyl­phenyl)-1-picrylhydrazyl (left) and the biradical (after Stuhrmann, 2023 ▸). Both molecules embedded in a deuterated medium R3 contain regions rich in hydrogen denoted by R2. Atoms close to the radical site belong to R1. The lower part of the figure shows the evolution of proton polarization in the reservoirs R1, R2 and R3. Proton polarization left: (blue) *P*_1_(*t*), (yellow) *P*_2_(*t*), (grey) *P*_3_(*t*); right: (blue) *P*_3_(*t*), (yellow) *P*_2_(*t*), (grey) *P*_1_(*t*). The reservoir of the outer protons in R2 and R3 is most distinct from R1 for the biradical (Stuhrmann, 2023 ▸).

Due to an initiative of Ben van den Brandt, two radical molecules of medium size were chosen to demonstrate the existence of intramolecular polarization gradients: 2,2-di(4-*tert*-octyl­phenyl)-1-picrylhydrazyl (loosely called DPPH) and the biradical with its two pyrrole rings (upper part of Fig. 4 ▸). The latter is a gift from Godt *et al.* (2000 ▸). Both have in common regions rich in hydrogen like the two octyls and the four hexyls, respectively. These compounds are stable at room temperature. The experiments were done under conditions of DNP (*i.e.B* = 3.5 T and *T* = 1 K). The direction of DNP was changed each 10 s. The microwave frequencies were 97.2 and 97.5 GHz for positive and negative proton polarization, respectively.

The analysis of time-resolved polarized neutron scattering in terms of equation (5) provides the evolution of the proton polarization in the reservoirs R1, R2 and R3. The transition probabilities 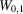, 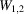 and 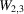, and the position of the intramolecular polarization gradient are chosen in such a way that the calculated *z*_3_(*Q*, *t*) of equation (5) fits the corresponding data from the scattering experiment: RMS defined by equation (7) is minimized (Stuhrmann, 2015 ▸, 2023 ▸). In the case of DPPH, the boundaries of significant electron spin density are known from magnetic neutron scattering, presented by the dotted line inside the DPPH molecule shown in Fig. 4 ▸ (Boucherle *et al.* 1982 ▸). The region of the polarized close protons of R1 of the same molecule is only slightly larger than that of polarized electron spin density.

Approximating the region of close protons by a sphere, the radius of R1 varies from 5 Å for pyrrole rings of the biradical to 7 Å for the picrylhydrazyl radical of DPPH. These results are essential for modelling the catalase molecule in terms of reservoirs.

## 4. Bovine liver catalase

The experiments with catalase are motivated by its enzymatic mechanism. Catalase is a redox enzyme, which converts hydrogen peroxide at a very fast, diffusion-limited rate into water and molecular oxygen. By offering per­oxy­acetic acid, a derivative of hydrogen peroxide with more steric hindrance to BLC, the reaction differs in at least two points: first, the response is slow; second, after some intermediate steps, one of its amino acids, tyrosine, is converted to a tyrosyl radical (Ivancich *et al.*, 1996 ▸, 1997 ▸). The number of tyrosyl radicals created in this way is low, typically less than one among the 500 amino acids of one subunit of the catalase molecule (Table 1 ▸).

The preparation of the radicalized catalase molecule is as follows. At a temperature only slightly above 0°C, to 1 ml of the clear solution of BLC dissolved in a mixture of equal volumes of glycerol-*d_n_* and heavy water, 28 µl per­oxy­acetic acid (32% solution, Merck, diluted to a 3% solution in 0.1 *M* Tris, and adjusted to pH 4.3) were added. The immediate formation of a porphyrin-π-cation radical and the subsequent slower formation of a tyrosyl radical can be followed by change of the colour to deep red. After a few minutes the tyrosyl concentration reaches its maximum and the sample is frozen to liquid nitro­gen temperature (Ivancich *et al.*, 1997 ▸). From the EPR measurements a tyrosyl concentration of 0.58 per heme has been determined.

**Figure 5:**
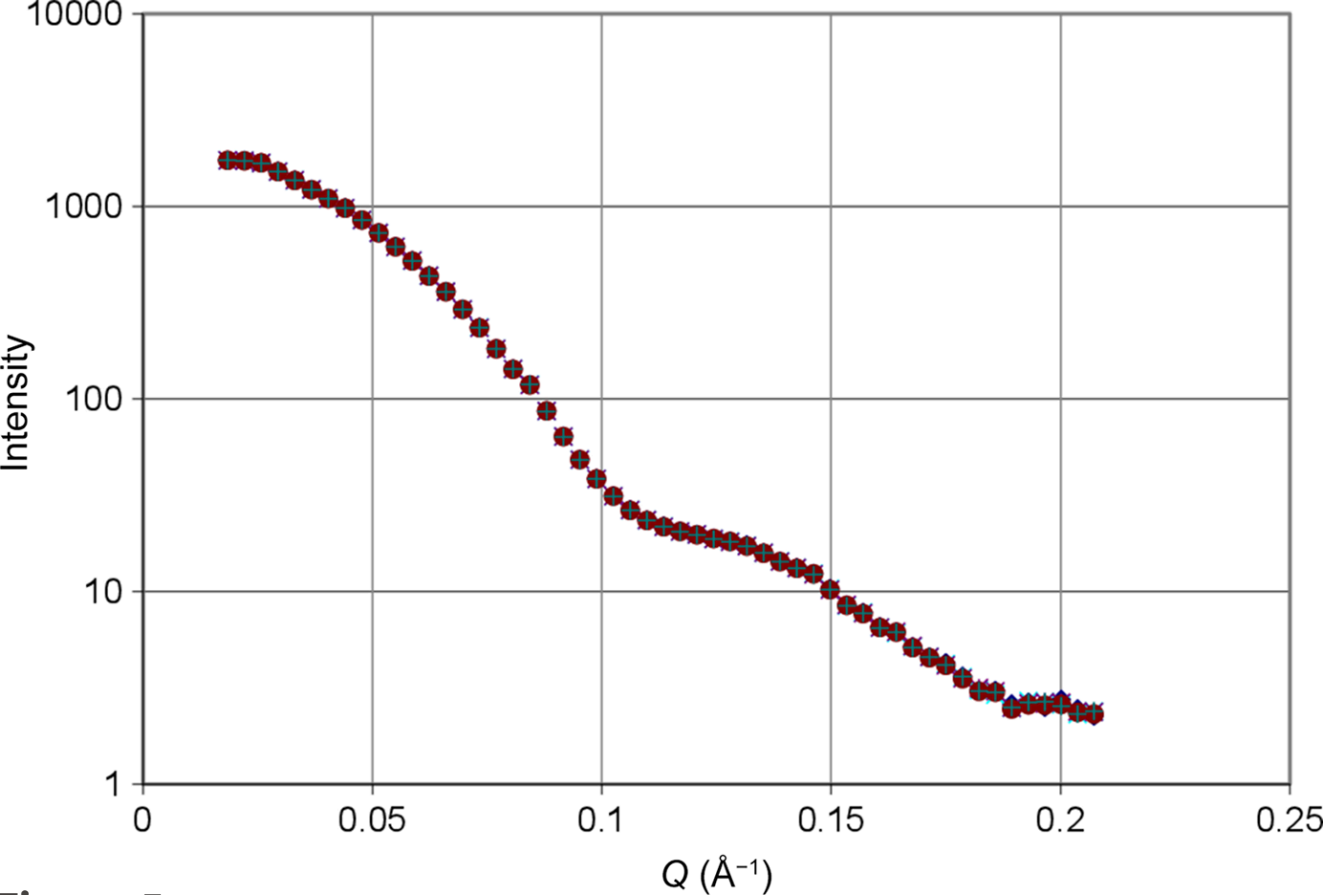
Neutron small-angle scattering of BLC dissolved in a mixture of equal volumes of deuterated glycerol and heavy water. Protein concentration: 32 g l^−1^; *T* = 1 K. This is the intensity collected during one cycle of 92 s duration. This cycle was repeated 70 times.

The intensity of small-angle scattering of BLC is shown in Fig. 5 ▸. The radius of gyration of the catalase molecule is *R*_g_ = 36 Å.

### 4.1. Polarization-dependent intensity of neutron scattering from BLC

Time-resolved neutron scattering from tyrosyl-doped catalase has been measured at six microwave frequencies close to the EPR profile of the tyrosyl, *i.e.* (1) below the EPR of tyrosyl: 97.15, 97.20 and 97.25 GHz; (2) above the EPR of tyrosyl: 97.45, 97.50 and 97.55 GHz; and a seventh one at *E* = 96.80 GHz assumed to be off-resonance. The peak of the EPR profile was assumed to be at 97.35 GHz.

**Figure 6:**
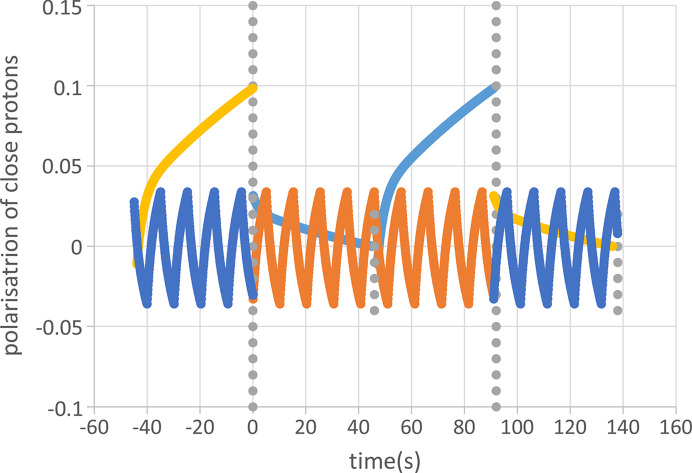
Representation of two sequences of close proton polarization. (1) The cycle of AFP-modulated polarization (blue) starts at *t* = 0 with a half-cycle of 46 s followed by 40 s of DNP and 6 s relaxation. At the end of each half-cycle (vertical lines of grey dots), the application of AFP leads to an important reduction of the polarization of the close protons that will be shown below in Fig. 12 ▸. This cycle is repeated 70 times. (2) The sequence of alternating directions of DNP (orange) works at a much faster rhythm of 10 s per period. The cycle is repeated several thousand times (Zimmer *et al.*, 2016 ▸).

An experiment of time-resolved neutron scattering from an AFP-modulated proton polarization at one of the frequencies mentioned above follows a rhythm described by the blue line in Fig. 6 ▸. A period of relaxation starting at *t* = 0 is interrupted by AFP after 46 s. Microwaves are switched on for another 40 s. After a short period of 6 s relaxation the cycle ends after 92 s with a second application of AFP.

To complement our data of time-resolved neutron scattering from AFP-modulated targets, we use data from another method relying on alternating direction of DNP induced by a periodic change of the microwave frequency as shown by an orange line in Fig. 6 ▸.

**Figure 7:**
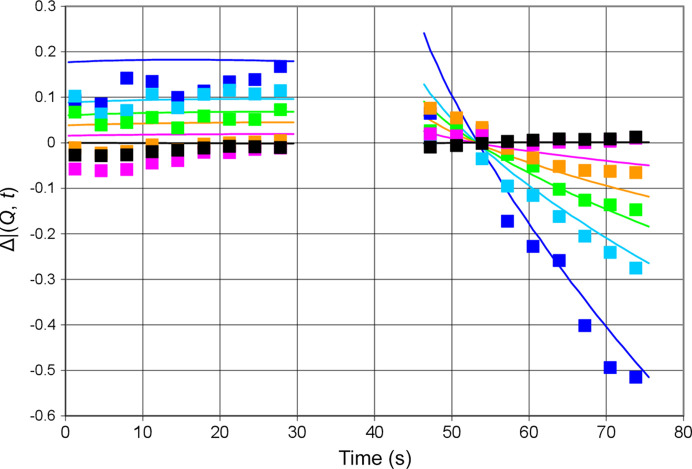
Intensity of neutron scattering versus time at various *Q* (Å^−1^): (dark blue) 0.040, (light blue) 0.059, (green) 0.066, (yellow) 0.073, (pink) 0.084, (black) 0.103. Lines are calculated using equation (9). The microwave frequency of 97.2 GHz leads to significant decrease of the intensity of neutron scattering during the second half-cycle due to a positive polarization of the protons inside the catalase molecule. Error bars are shown for *Q* = 0.040 and 0.073 Å^−1^.

A typical result of time-resolved neutron scattering including AFP is shown in Fig. 7 ▸. The polarization-dependent data are on the same scale as in Fig. 5 ▸. With about one per every thousand of the total neutron scattering intensity they are very noisy. The polarization-dependent part is obtained as the difference between the total intensity of small-angle scattering *I*(*Q*, *t*) and its time average. For the experimental data we have
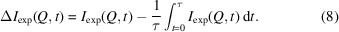
Similarly for the calculated data, it holds that
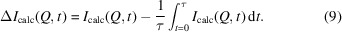
For *I*_calc_(*Q*, *t*), see equation (6), with *Z*_3_(*Q*, *t*) replaced by *Z*_5_(*Q*, *t*).

There is a decrease of the intensity of neutron scattering during the second half-cycle due to a dynamic polarization of the protons into the positive direction. Keep in mind that we use a deuterated solvent with a relatively high scattering density which largely surpasses that of the hydrogenous catalase molecule. An increase of the scattering density of the solute lowers the contrast of the protein with respect to the deuterated solvent and hence its neutron scattering intensity shown in Fig. 7 ▸.

The following figures of time-resolved neutron scattering contain, in addition to the experimental data, lines presenting 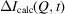 which, rather than being a guide for the eye, are also a fit to these data based on a model of the catalase molecule in terms of five reservoirs presented in Section 4.2.

**Figure 8:**
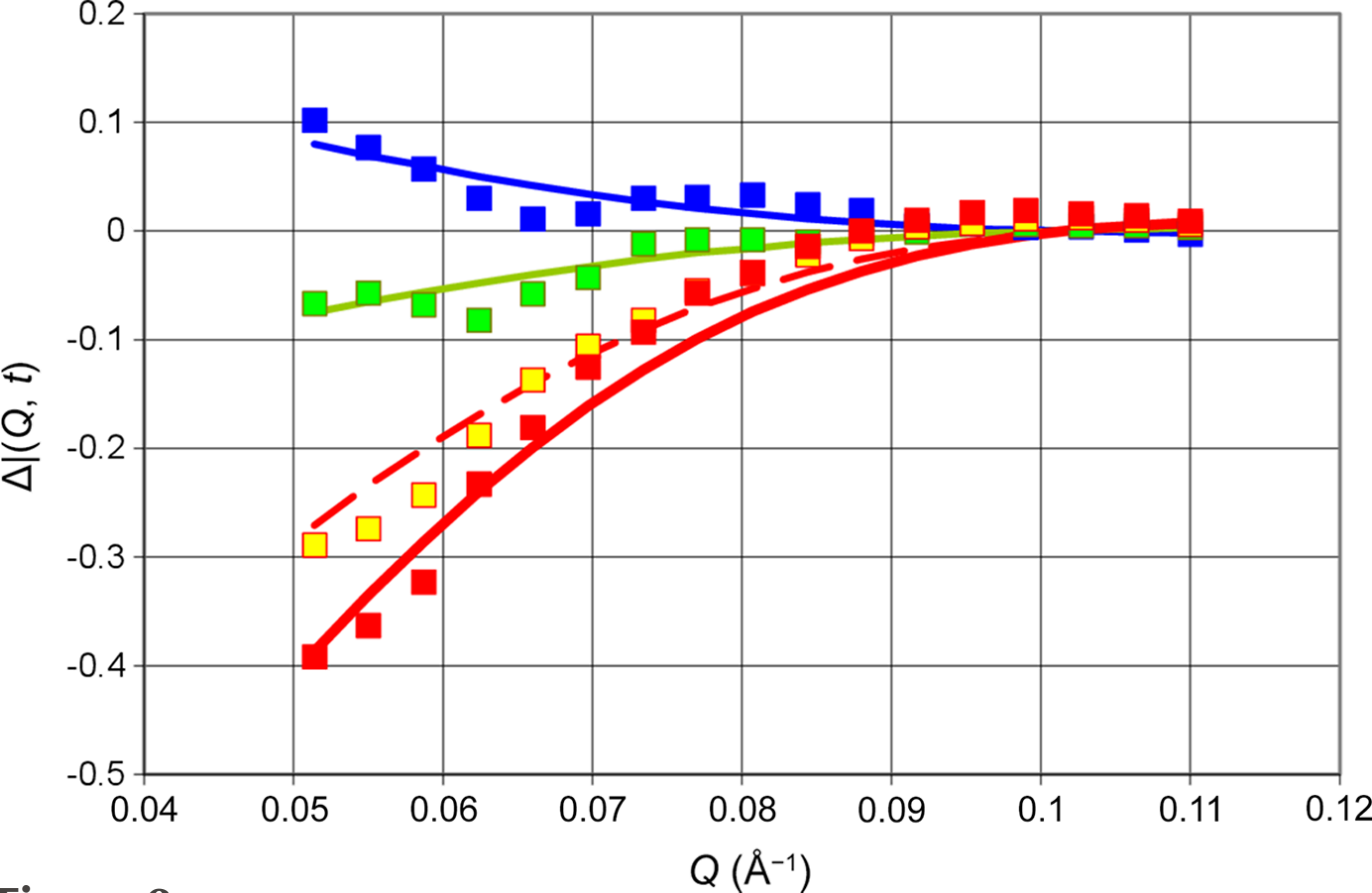
Neutron scattering intensity versus *Q* (Å^−1^) at various times: (blue) 50 s, (green) 57 s, (yellow) 67 s and (red) 73 s. Squares present experimental data defined by equation (8). Lines are calculated using equation (9). Frequency 97.2 GHz. Error bars are shown for 73 s at *Q* = 0.053 and 0.081 Å^−1^. They do not vary with time.

Another view of the results presented in Fig. 7 ▸ is shown in Fig. 8 ▸. The intensity of neutron small-angle scattering versus *Q* changes during the half-cycle of DNP. At the onset of DNP, the polarization-dependent intensity is positive. After 73 s it will have become strongly negative.

A similar dispersion of DNP is found at microwave frequencies of 97.15 and 97.25 GHz, both giving rise to dynamic polarization in the positive direction.

At this point, it is appropriate to comment on the treatment of the experimental data. The intensity shown in the figures is a local average. At *t_i_* and *Q_j_* the sum covers values from *i* = −8 to *i* = +8 and *j* = −4 to *j* = +4, respectively. The originally non-linear time intervals have been linearized to equal intervals of 0.83 s.

**Figure 9:**
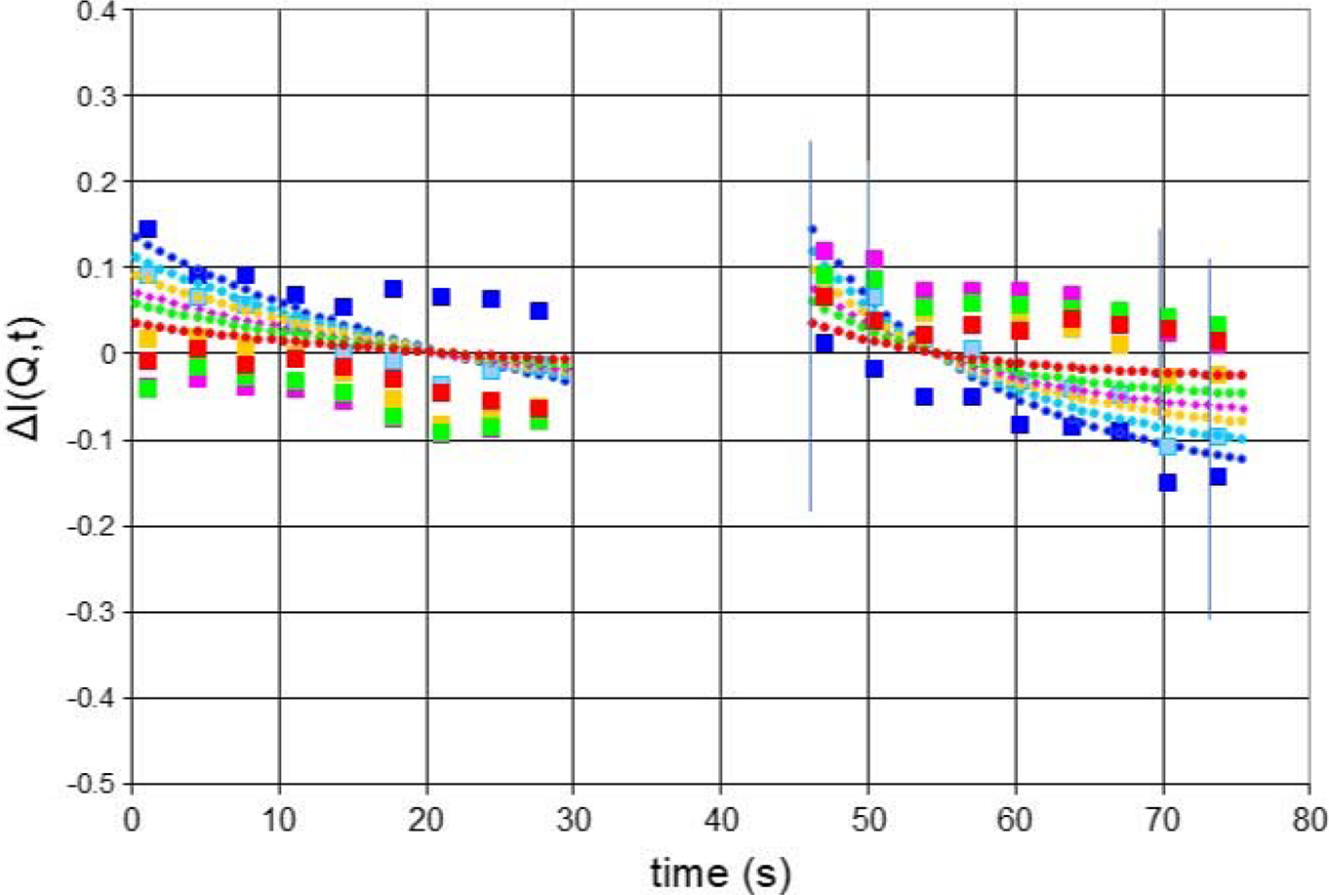
Intensity of neutron scattering versus time at various *Q* (A^−1^): (dark blue) 0.051, (light blue) 0.055, (yellow) 0.059, (pink) 0.062, (green) 0.066, (red) 0.073, (black) 0.081. Squares present measured intensities as defined by equation (8). Lines are calculated using equation (9). Frequency 97.50 GHz. Error bars are shown for 0.0.051 and 0.073 Å^−1^.

**Figure 10:**
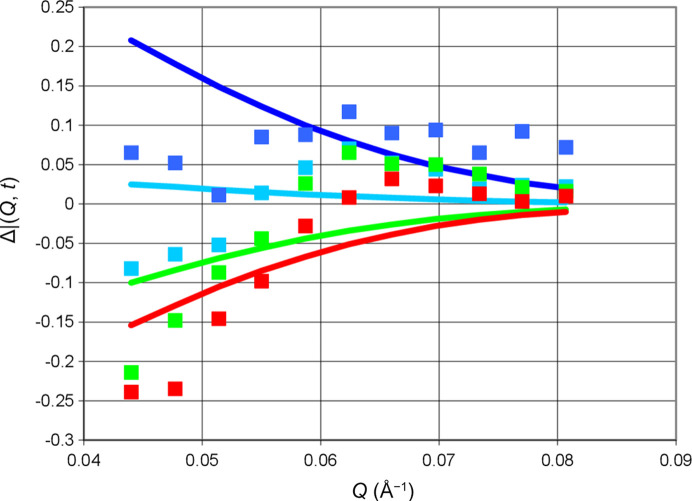
Neutron scattering intensity versus *Q* (Å^−1^) at various times: (dark blue) 47 s, (light blue) 53 s, (green) 63 s and (red) 73 s. Squares present polarization-dependent experimental data as defined by equation (8). Lines are the data calculated using equation (9). Frequency: 97.50 GHz. Error bars are shown for 0.048 and 0.073 Å^−1^. They do not vary with time.

Let us turn to a microwave frequency that is expected to give rise to negative proton polarization, say 97.5 GHz. The change of the neutron scattering during one half-cycle of DNP is smaller by a factor of 3. More surprisingly, there is a decrease of the scattering intensity during both half-cycles (Fig. 9 ▸). Is the proton polarization really increasing? In fact, it is. The drift of proton polarization towards values close to its thermal equilibrium, at *P*_e_ = 0.35%, is sufficient to outclass the influence of DNP in the negative direction. Fig. 9 ▸ shows the change of the intensity of neutron small-angle scattering with time during the half-cycle of DNP. The intensity of small-angle scattering at various times is shown in Fig. 10 ▸.

A similar picture is observed at energies of 97.45 and 97.55 GHz. Is there still a trace of negative DNP at frequencies slightly above the EPR? An answer is expected from a more profound analysis of our experiment.

### 4.2. The reservoirs of the catalase molecule

The catalase molecule has a molecular weight of *M* = 240 000 or 240 kDa (kilodalton). Catalase is a tetramer molecule. It consists of four identical subunits with *M* = 60 kDa each. Each subunit is made of about 500 amino acids; 20 of them are tyrosine. One of the tyrosines may be converted into a tyrosyl radical. Its R1 is 0.5 kDa, less than 1% of the subunit. We used the structure of catalase obtained from X-ray crystal diffraction (Fita & Rossmann, 1985 ▸).

At the onset of DNP, a few polarized protons will find themselves in an ocean of unpolarized protons. The initially localized proton polarization will spread out. In view of the large space left inside the catalase molecule it would be conceivable to introduce a second reservoir as a shell surrounding a spherical R1 of 10 Å diameter (Zimmer *et al.*, 2016 ▸). An R2 of 20 Å diameter would also comply with findings from NMR experiments of Wolfe (1973 ▸) aiming for direct observation of a nuclear spin diffusion barrier.

Each of the four subunits of the catalase molecule contains one heme group. As it presents a kind of magnetic inhomogeneity it might influence the migration of the proton polarization towards the molecular surface. The four iron atoms of the heme are roughly on a surface of a sphere of 60 Å diameter, centred at the midpoint of the total molecule.

On average, we have *N_i_* protons in equations (10) and (11) shown below in each reservoir *Ri* as follows.

R1, very close protons: 100;

R2, not so close protons: 500;

R3, protons inside the magnet trap (*r* = 30 Å): 3200;^1^

R4, residual protons of the catalase molecule: 8700; and

R5, sparce protons of the deuterated solvent: 40 000.^2^

**Figure 11:**
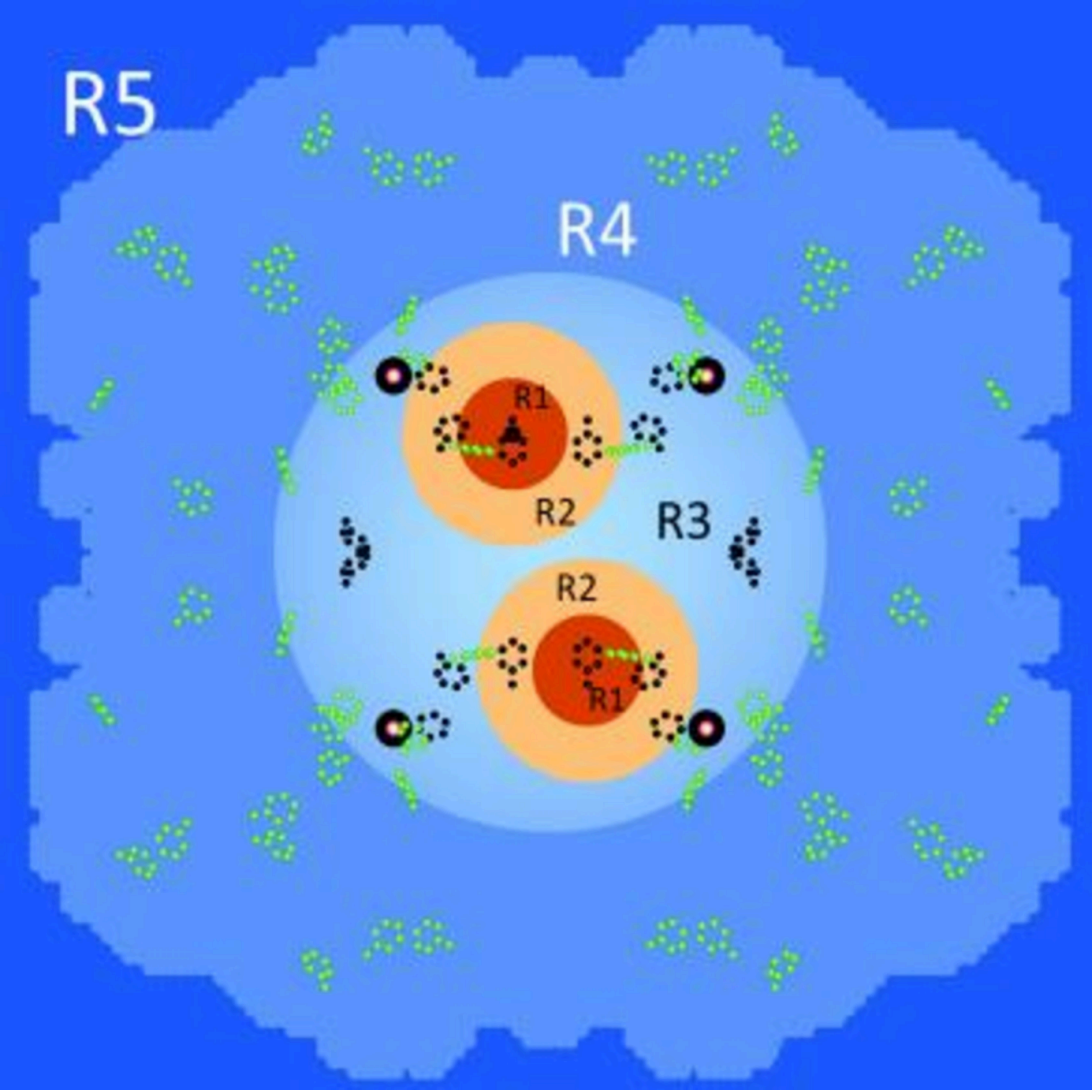
The five reservoirs of the tetramer catalase molecule and its tyrosines (shown by their phenyl rings, in light green). The reservoir R3 includes the four heme groups presented by their iron atoms and the potential tyrosyls (dark blue) as proposed by Zimmer *et al.* (2016 ▸). The red and orange spheres are centred at the sites of Tyr369. The reservoirs in blue are assumed to be accessible to AFP.

A model of the catalase molecule quite similar to that shown in Fig. 11 ▸ consisting of four reservoirs has been used for a first localization of the tyrosyl molecule (Zimmer *et al.*, 2016 ▸). An additional spherical proton spin diffusion barrier with a radius of 30 Å centred at the midpoint of the catalase molecule has been introduced into the present model. We take it for granted in the following evaluation of the proton polarization in each of the five reservoirs.

In a first step, we will compare the experimental intensities of time-resolved neutron scattering with the corresponding data calculated from the model. The variables used for the fit are:

(1) Transition probabilities *W*_*i*,*j*_ [see equations (10) and (11)].

(2) Efficiency of the reservoir-dependent AFP, supported by Fig. 2 ▸.

(3) Influence of the drift of proton polarization to thermal equilibrium at *P*_e_.

(4) Intensity at the onset of microwave irradiation in R1 refined by more precise data from experiments using alternating directions of DNP (Zimmer *et al.*, 2016 ▸).

We extend equation (2) to five reservoirs R1 to R5. For the leading four reservoirs the coupled differential equations are
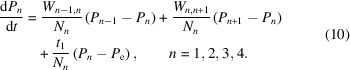
The proton polarization in the fifth reservoir is written as
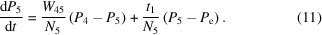
Equation (5), defining *Z*_3_(*Q*, *t*), is then extended to
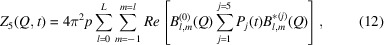
where 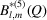 is the amplitude of the solvent protons contained inside the shape of the catalase molecule. As such, its sign is negative. *P*_5_(*t*) is the time-dependent polarization of the solvent.

**Figure 12:**
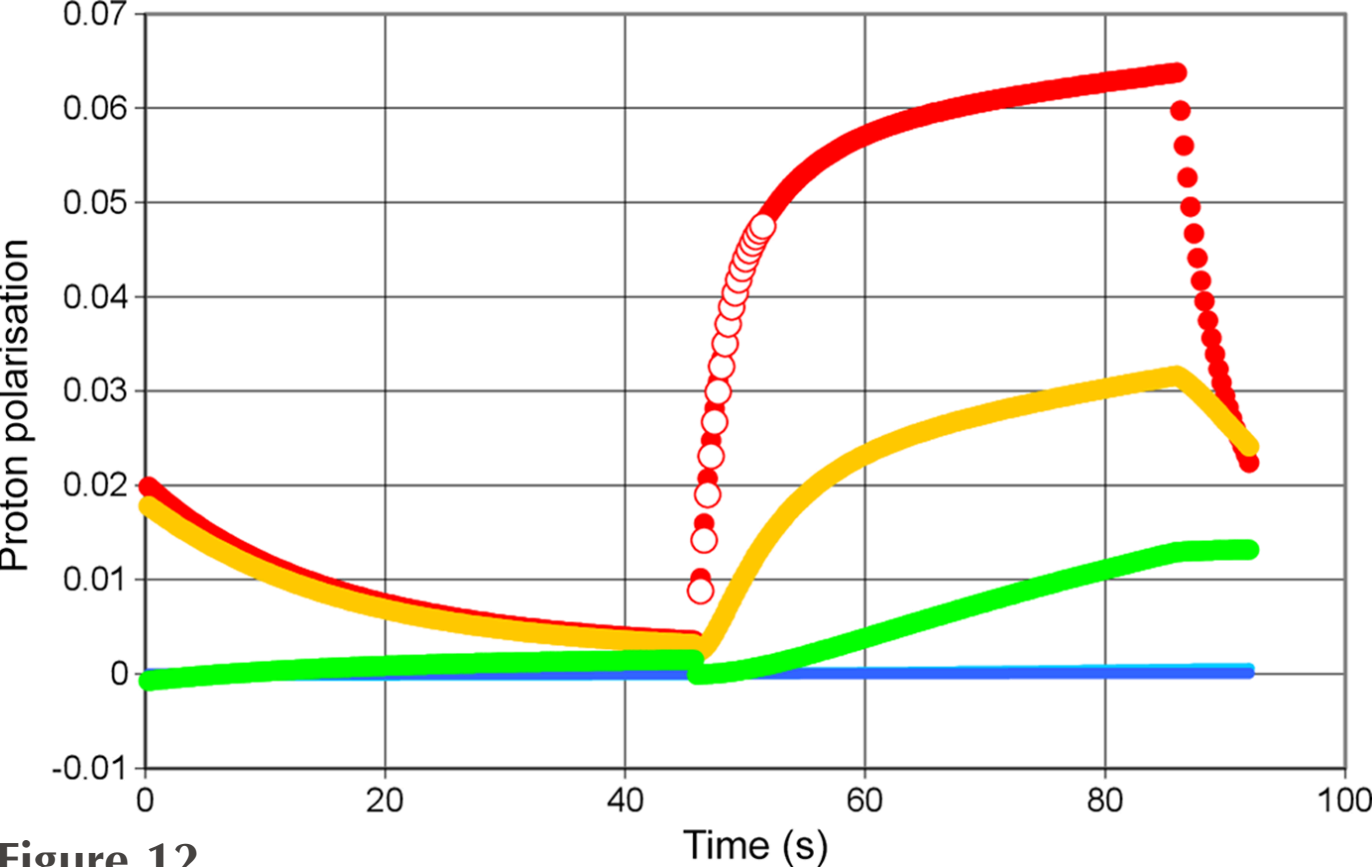
Polarization of the protons in the reservoirs R1 to R5. The direction of DNP is positive. (Red, filled circles) R1, (yellow, filled circles) R2, (green, filled circles) R3, (light blue) R4, (dark blue) R5, (red, unfilled circles) R1 from method using alternating direction of DNP [point (4) mentioned above]. The method of AFP is applied at 46 s and 92 s. *E* = 97.20 GHz.

The drift of the polarization towards thermal equilibrium at *P*_e_ = 0.35% is taken into account by *t*_1_, which in a way may be identified with the spin lattice relaxation time *T*_1_ shown in equations (10) and (11). High values of *t*_1_ are encountered with negative DNP exceeding those at positive DNP by a factor of 10. These results are obtained from comparing the calculated intensity defined by equation (9) with the corresponding experimental data defined by equation (8),[Disp-formula fd8]
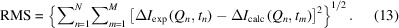
The results shown in Figs. 12 ▸, 13 ▸ and 14 ▸ are obtained by minimization of RMS in equation (13).

Using a microwave frequency of 97.2 GHz we observe a rapid increase of the polarization of the close protons in R1 to *P*_1_(*t*) = 0.06, after 40 s of DNP. Following point (4) mentioned above, the evolution of proton polarization is guided by results from the method using alternating directions of DNP (red circles in Fig. 12 ▸). There is still a significant polarization of the protons in R2 and R3. Beyond R3 there is a dramatic drop of proton polarization to values close to zero. The boundaries of R3 include the iron atoms of the heme (Fig. 11 ▸). A justification of R3 is given in Section 5.

The efficiency of AFP is strongly dependent on the reservoir. It is highest in the solvent region R5 where ɛ = 0.4. The outer protons of catalase with ɛ = 0.3 are only slightly more reluctant to follow AFP. Inside the barrier defined by the heme, the signal of AFP is hardly heard: ɛ = 0.05. According to equation (1), ɛ = 0 would correspond to complete depolarization of the proton spin system. Inside R2 and R1 the efficiency ɛ of AFP becomes negative. A small part of the proton spins will be affected by AFP. An estimate of this fraction appears to be around 6% (Leymarie, 2002 ▸).

The data for time-resolved neutron scattering at microwave frequencies of 97.15 and 97.25 GHz provide similar results.

**Figure 13:**
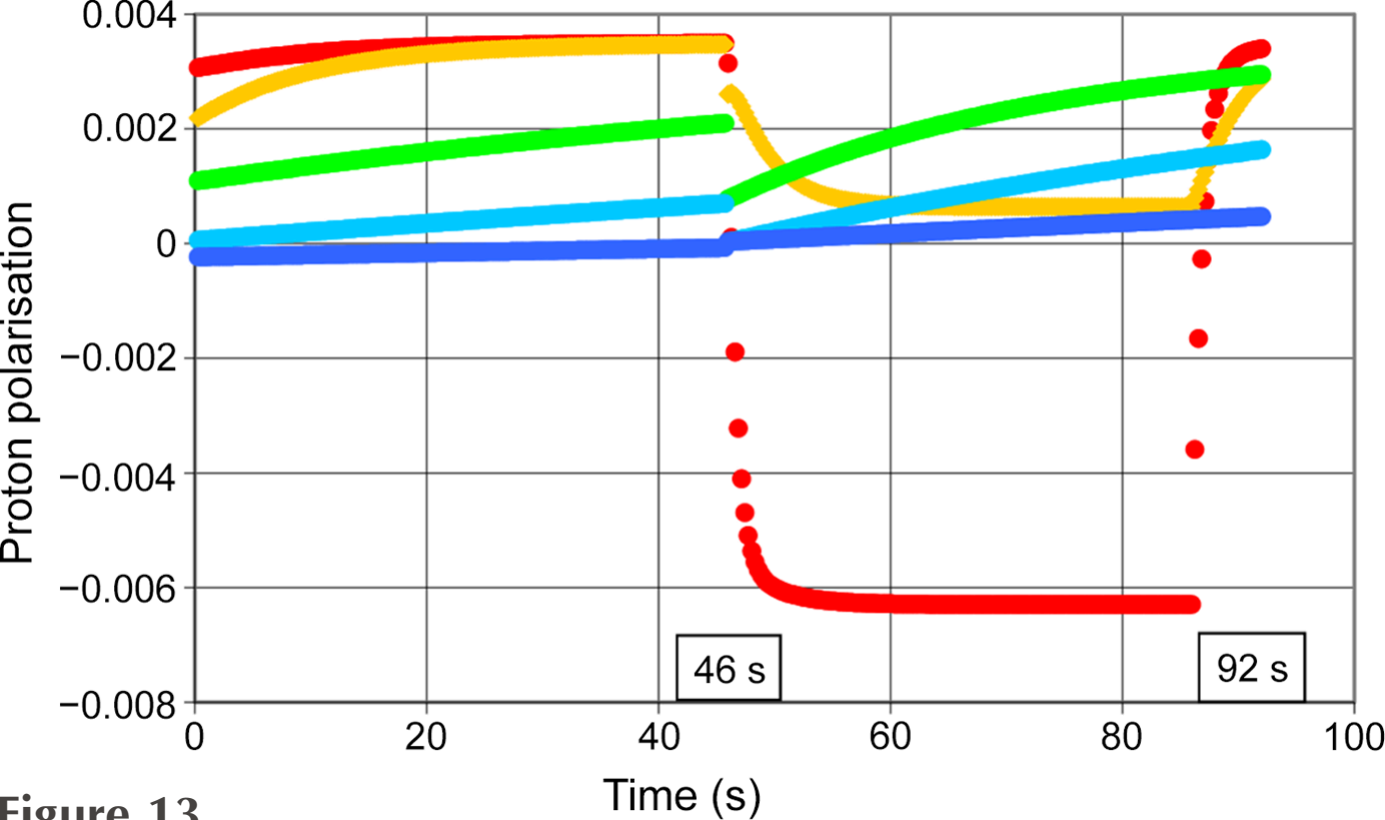
Polarization of the protons in the reservoirs R1 to R5. The direction of DNP is negative. The method of AFP has been applied at 46 s and 92 s. Microwave frequency 97.50 GHz. Symbols are the same as in Fig. 12 ▸.

Now let us look at DNP in the negative direction. Fig. 13 ▸ shows a very weak negative polarization of the protons in R1. The polarization ends at *P*_1_(*t*) = − 0.006 after a few seconds of microwave irradiation. There is hardly any transfer of polarization to the subsequent reservoirs. In the absence of DNP-controlled proton polarization outside R1, the movements of polarization are governed by spin lattice relaxation and periodic proton spin reversion by AFP.

**Figure 14:**
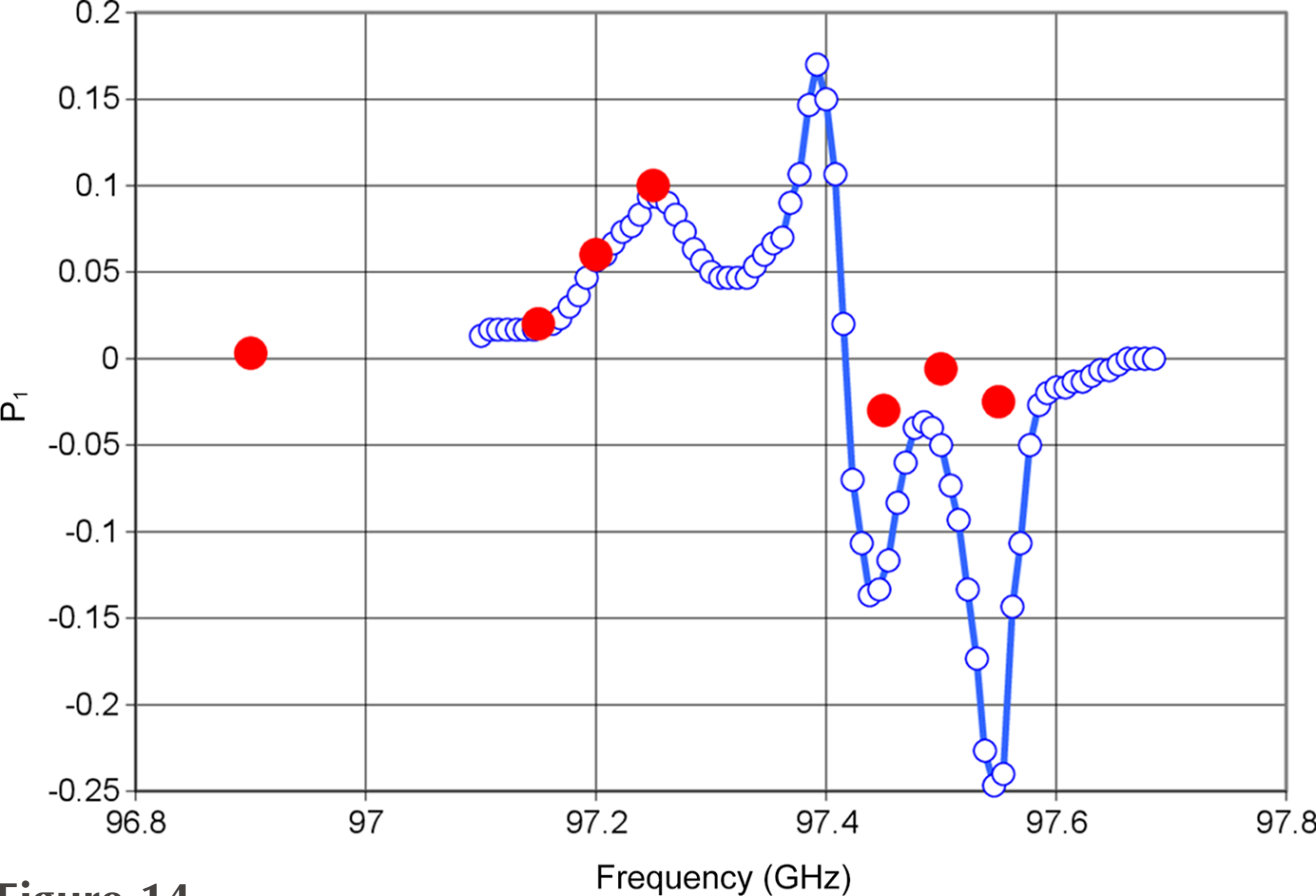
Polarization of protons in R1 achieved by DNP (red dots) at frequencies below the EPR follows the EPR line. The latter is in arbitrary units. This is less visible at frequencies above the EPR line where the effect of DNP becomes weak. The estimated vertical error bar may slightly exceed the diameter of the red circles.

The efficiency of DNP appears to follow the line of EPR (Fig. 14 ▸). This is most striking for the microwave frequencies 97.15, 97.20 and 97.25 GHz. At frequencies leading to DNP in the negative direction even the very low efficiency DNP fits the EPR line remarkably well.

## 5. The justification of the structural parameters

The model shown in Fig. 11 ▸ contains two peculiarities which so far were not substantiated by a direct observation. They concern: (1) the location of the tyrosyl radical inside the catalase molecule; (2) the diffusion barrier defining the reservoir R3.

A first attempt to locate the tyrosyl radical has already been undertaken by Zimmer *et al.* (2016 ▸). Although it remained to some extent ambiguous the result was compatible with findings from a detailed analysis of the EPR line (Ivancich *et al.*, 1996 ▸, 1997 ▸; Svistunenko & Cooper, 2004 ▸). In fact, EPR lines from the tyrosyl radical are sensitive to the orientation of their phenyl group with respect to the backbone of the amino-acid chain. The localization of the radical at Tyr369 relies on both the EPR line and a high-resolution structure of the protein from X-ray crystallography.

**Figure 15:**
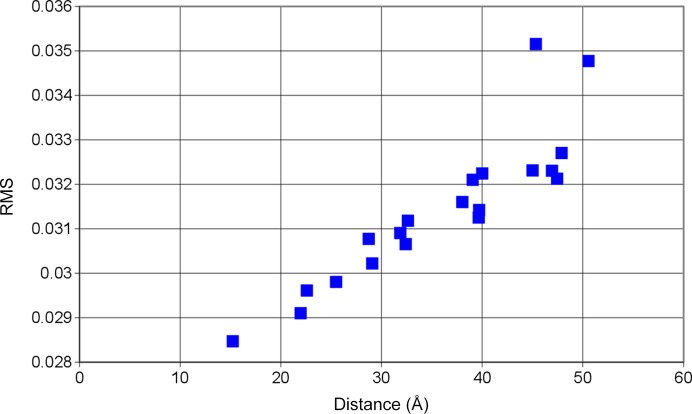
RMS deviation between the calculated and measured neutron scattering intensity for 20 tyrosines of the BLC subunit, located at distances between 15 and 50 Å from the centre of the tetrameric catalase molecule. Notably, Tyr369, the one closest to the centre, was converted into a tyrosyl radical. Data for positive DNP at a microwave frequency of 97.2 GHz were utilized.

In our present approach applying the method of AFP to a system of dynamic polarized protons the result appears to be quite straightforward (Fig. 15 ▸). It is the tyrosine number 369 of the amino-acid chain that has been converted into a radical.

The deviation of the calculated 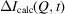 from the experimental time-resolved neutron scattering intensity 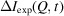 has been determined for each of the 20 tyrosines of the catalase subunit located at distances between 15 and 50 Å from the centre of the tetramer catalase (Fig. 15 ▸).

It happens that the Tyr369 at a distance of 15 Å from the centre of the catalase molecule has been converted to a tyrosyl radical. The distance between the four potential tyrosyl sites of the total catalase molecule is only around 25 Å. With an occupancy of 0.58 the tyrosyls per heme might often find themselves crowded close together.

The idea of an intermediate boundary defined by the surface of a sphere with the radius of R3 emerged from the inspection of the projection of the catalase structure shown in Fig. 11 ▸. With this projection of the catalase molecule, the iron atoms of the four hemes appear to be close to a circle defining R3.

**Figure 16:**
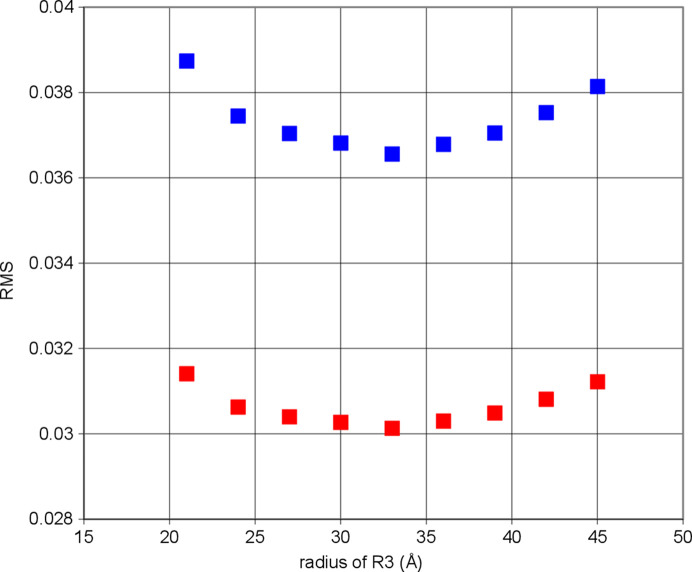
The radius of R3 has been varied between 21 Å and 45 Å; the best agreement between calculated and experimental data is obtained with *r* = 33 Å as the radius of R3. The RMS is based on two different *Q* intervals: (blue) 0.058 Å^−1^ < *Q* < 0.106 Å^−1^ and (red) 0.058 Å^−1^ < *Q* < 0.135 Å^−1^. Data for positive DNP at a microwave frequency of 97.2 GHz were utilized.

The justification of R3 proceeds in a way which is quite similar to that used for the location of the radical. The RMS is calculated for a number of possible radii of R3 (Fig. 16 ▸). The best fit with the experimental data is obtained with a radius close to 33 Å. R3 then contains the iron atoms of the four heme moieties. Although this result is plausible, the model of a spherical reservoir R3 is certainly a simplification.

This not quite unexpected finding gives rise to a number of questions. What is the nature of the magnetic trap mentioned above? Although the vicinity of R3 to the iron atoms of the heme group is intriguing, the nature of the presumably magnetic inhomogeneity is an open question.

In spite of the high noise level of the experimental data, the application of the mathematical formalism presented above appears to be a powerful filter. The radical site of Tyr369 known from EPR studies has been confirmed by our approach. Moreover the detection of a kind of magnetic inhomogeneity perturbing the flow of proton polarization emerging from the excited radical site is new and will need more in-depth studies.

The determination of the radical site and the radius of the sphere of magnetic turbulence are independent of the method of data reduction. In this paper, we used local averages in the space of *Q* and time *t*. This allows an easy retrieval of the statistical error.

Other ways of data reduction relying on analytical functions have also been tried. The RMS defined by equation (13) utilizing the reduced data set then is considerably lower. The analytical approximations provide the same results that have been reported in this paper.

## 6. Conclusions

The selective inversion of nuclear spin polarization via the AFP method allows for precise tailoring of the spatial distribution of dynamically polarized protons. This approach introduces a new dimension of DNP by complementing the conventional method which primarily targets the source of the polarization contrast. AFP exerts its full effect on nuclear spins located far from the radical sites responsible for DNP while leaving protons near the unpaired electrons largely unaffected. The strategy of combination of AFP and DNP, applied in a carefully designed sequence that includes relaxation, provides optimal conditions for kinetic and structural studies. This methodology is particularly valuable for investigating both active centres supporting DNP and inactive regions, such as magnetic inhomogeneities in complex structures like radical proteins using time-resolved polarized neutron scattering.

## Figures and Tables

**Table 1:** Concentration of unpaired electrons in tyrosyl-doped catalase

Sample	No. of unpaired electrons per molecule	Unpaired electrons per cm^3^
Bovine liver catalase, 4 equal subunits, 60 kDa molecular weight each†	Up to 4 (*i.e.* up to 1 per heme)	1.8 × 10^17^ = 300 µ*M* l^−1^ (occupancy 0.58)‡

† Each contains one iron bound to a protoheme IX group. Solvent: 1:1 glycerol-*d_n_*:D_2_O.

‡ 500 µ*M* l^−1^ heme equals 32 g l^−1^ catalase.

## Footnotes

1 *N*_3_ + *N*_4_ = 11 900.

2 Only the protons of the solvent displaced by the volume of the catalase molecule, *i.e.* 700 out of *N*_5_ = 40 000, contribute to the partial amplitude  in equation (12).
